# P-1184. Efficacy and Safety of Imipenem/Cilastatin/Relebactam (IMI/REL) in Treating Hospital-Acquired and Ventilator-Associated Bacterial Pneumonia: A Systematic Literature Review of Randomized Control Trials

**DOI:** 10.1093/ofid/ofaf695.1377

**Published:** 2026-01-11

**Authors:** Carolyn Cameron, Shalini Bagga, Vaneet Pal Kaur Khurana, Prashant Soni, Sachin Kumar, Emre Yucel

**Affiliations:** MSD Australia, Sydney, New South Wales, Australia; CHEORS LLC, Chalfont, Pennsylvania; CHEORS LLC, Chalfont, Pennsylvania; CHEORS LLC, Chalfont, Pennsylvania; CHEORS LLC, Chalfont, Pennsylvania; Merck & Co., Inc., North Wales, PA

## Abstract

**Background:**

Pneumonia, a serious lung infection caused by bacteria, viruses, or fungi, often results in severe complications such as acute respiratory failure and sepsis, especially in ICU settings with high mortality rates. Hospital-acquired bacterial pneumonia (HABP) and ventilator-associated bacterial pneumonia (VABP) are notable forms, frequently caused by multidrug-resistant (MDR) bacteria like Pseudomonas aeruginosa, Klebsiella, and methicillin-resistant Staphylococcus aureus. We aimed to conduct a systematic literature review (SLR) of randomized control trials (RCTs) published up to July 15, 2024 of IMI/REL to evaluate its efficacy and safety for treating HABP/VABP compared to other interventions.

**Figure 1.**
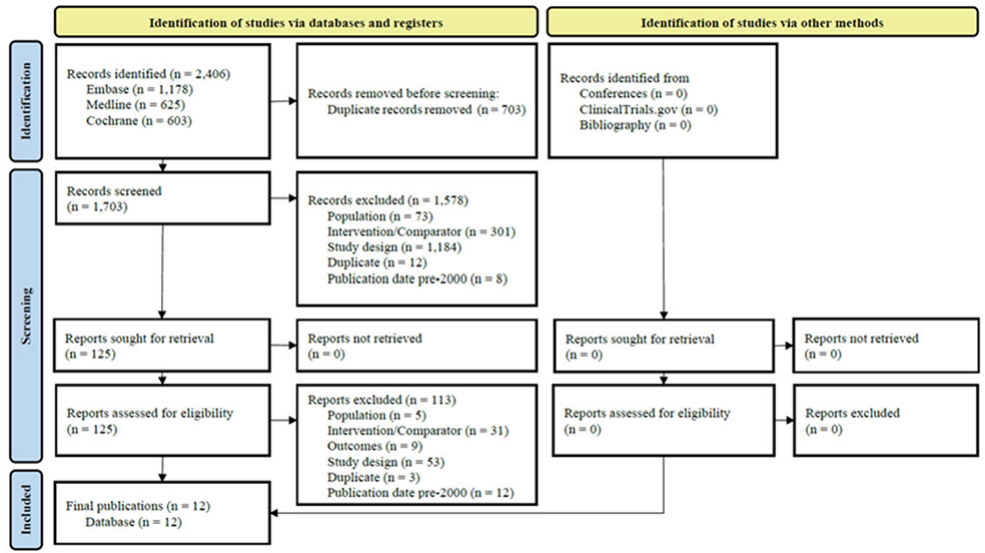
Flow diagram of study selection according to PRISMA guidelines

**Figure 2.**
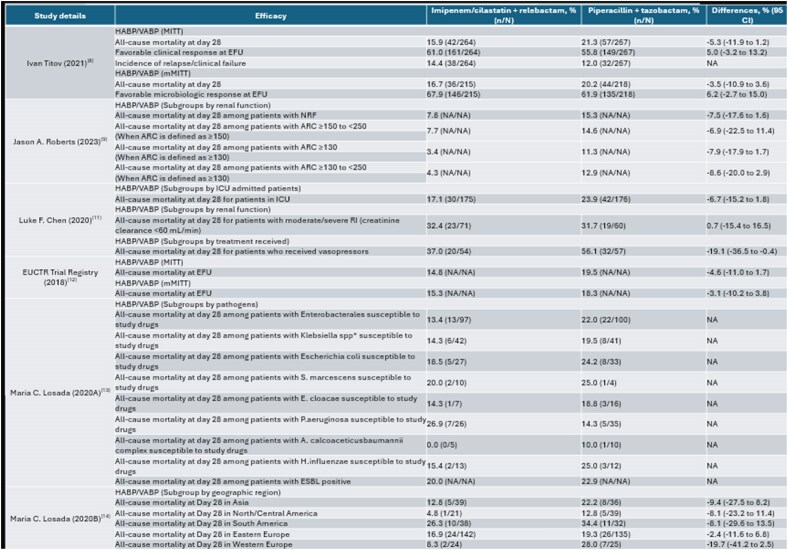
Efficacy of IMI/REL Abbreviations: ARC: Augmented Renal Clearance; APACHE II: Acute Physiology and Chronic Health Evaluation II; CI: Confidence Interval; EFU: Early Follow-Up; EOT: End of Treatment; ESBL: Extended-Spectrum Beta-Lactamase; ICU: Intensive Care Unit; h: Hour; HABP: Hospital-Acquired Bacterial Pneumonia; MITT: Modified Intent-to-Treat; mMITT: Microbiological Modified Intent-to-Treat; mL/min: Milliliters Per Minute; NA: Not Applicable; NRF: Normal Renal Function; RI: Renal Impairment; SmMITT: Supplemental Microbiological Modified Intent-to-Treat; spp: Species; VABP: Ventilator-Associated Bacterial Pneumonia *Klebsiella spp. included Klebsiella aerogenes, Klebsiella oxytoca, and Klebsiella pneumoniae. Note: Only unique data reported for each study in the table above. Any results from primary publications that were repeated in secondary publications were considered duplicates and, therefore, not reported. Data for the APACHE score subgroup from the Ignacio Martin-Loeches (2023)(12) study was already reported in the primary publication; therefore, it was not included in the table above.

References in the table:

8. Titov I, Wunderink RG, Roquilly A, Rodríguez Gonzalez D, David-Wang A, Boucher HW, et al. A randomized, double-blind, multicenter trial comparing efficacy and safety of imipenem/cilastatin/relebactam versus piperacillin/tazobactam in adults with hospital-acquired or ventilator-associated bacterial pneumonia (RESTORE-IMI 2 Study). Clinical Infectious Diseases. 2021;73(11):e4539-e48.

9. Roberts JA, Nicolau DP, Martin-Loeches I, Deryke CA, Losada MC, Du J, et al. Imipenem/cilastatin/relebactam efficacy, safety and probability of target attainment in adults with hospital-acquired or ventilator-associated bacterial pneumonia among patients with baseline renal impairment, normal renal function, and augmented renal clearance. JAC-Antimicrobial Resistance. 2023;5(2):dlad011.

10. Martin-Loeches I, Shorr AF, Kollef MH, Du J, Losada MC, Paschke A, et al., editors. Participant-and Disease-Related Factors as Independent Predictors of Treatment Outcomes in the RESTORE-IMI 2 Clinical Trial: A Multivariable Regression Analysis. Open Forum Infectious Diseases; 2023: Oxford University Press US.

11. Chen LF, Losada MC, Mahoney KA, Du J, Brown ML, Tipping R, et al., editors. 1460. Imipenem/Cilastatin (IMI)/Relebactam (REL) in Hospital-Acquired/Ventilator-Associated Bacterial Pneumonia (HABP/VABP): Subgroup Analyses of Critically Ill Patients in the RESTORE-IMI 2 Trial. Open Forum Infectious Diseases; 2020: Oxford University Press US.

12. EUCTR. IMI/REL (MK-7655A) vs. PIP/TAZ in Treatment of Subjects with HABP/VABP. European Union Clinical Trials Register. 2018.

13. Losada MC, Maniar A, Du J, Brown ML, Young K, Hilbert DW, et al., editors. 1230. Clinical and Microbiologic Outcomes by Causative Pathogen in Hospital-Acquired or Ventilator-Associated Bacterial Pneumonia (HABP/VABP) Treated with Imipenem/Cilastatin (IMI)/Relebactam (REL) Versus Piperacillin/Tazobactam (PIP/TAZ). Open Forum Infectious Diseases; 2020: Oxford University Press.

14. Losada MC, Du J, Brown ML, Young K, Moise P, Tipping R, et al., editors. Efficacy and safety of imipenem/cilastatin/relebactam versus piperacillin/tazobactam in patients with hospital-acquired bacterial pneumonia/ventilator-associated bacterial pneumonia by geographic region. Intensive Care Medicine Experimenta; 2020.

15. Clinicaltrial.gov. Imipenem/Cilastatin/Relebactam (MK-7655A) Versus Piperacillin/Tazobactam in Participants With Hospital-Acquired or Ventilator-Associated Bacterial Pneumonia (MK-7655A-016). Clinicaltrialgov registry. 2018.

**Methods:**

PICOTS included patients aged ≥18yrs, requiring hospitalizations and treatment for a bacterial infection for HABP/VABP and receiving IMI/REL (Table 1). Systematic literature searches were conducted in Medline, Embase, and Cochrane databases up to July 15, 2024. Additionally, relevant conferences, HTA websites, and bibliographies of identified systematic reviews were hand-searched. Study selection involved two independent reviewers, with conflicts resolved by a third reviewer. Quality assessment was conducted using the Cochrane Risk-of-Bias Tool.

**Table 1.**
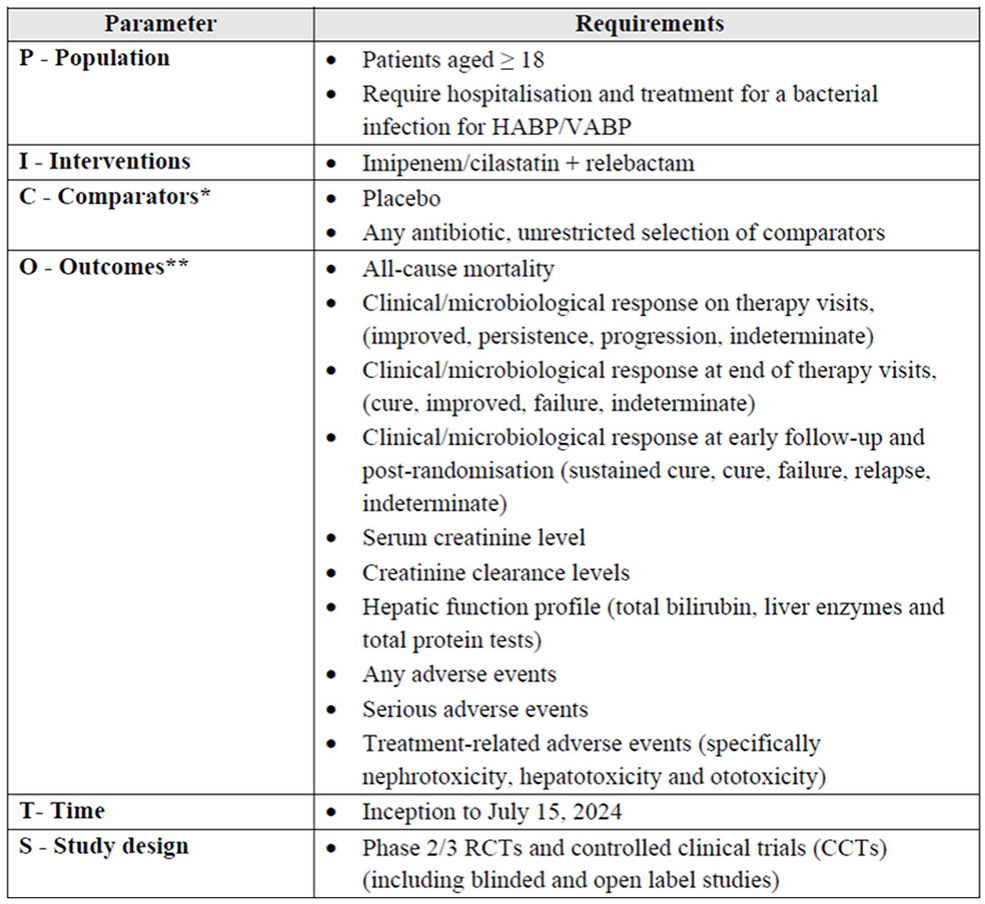
PICOTS

**Results:**

From 2,406 database records, 12 publications were included after title/abstract and full-text screening (Figure 1). These encompassed three unique trials: RESTORE-IMI 1, RESTORE-IMI 2, and NCT03583333. The trials consistently demonstrated the efficacy of IMI/REL in all-cause mortality and clinical responses for HABP/VABP. The safety profile of IMI/REL was comparable (Figure 2).

**Conclusion:**

The SLR provides strong evidence of IMI/REL's efficacy and safety for treating HABP/VABP, particularly benefiting critically ill patients, including those with severe infections and compromised renal function as shown in the RESTORE-IMI-1 and 2 trials. Facing significant multidrug-resistant (MDR) challenges, imipenem/relebactam may be effective where pathogens may be resistant to existing therapies. These findings will contribute to health technology assessments and facilitate integration of IMI/REL into treatment guidelines in additional geographic regions.

**Disclosures:**

Carolyn Cameron, MPH, MSD: Stocks/Bonds (Public Company) Shalini Bagga, TBD, Amgen: Work in a consultancy that works with Pharma companies|BI: Work in a consultancy that works with Pharma companies|BMS: Work in a consultancy that works with Pharma companies|Merck: Work in a consultancy that works with Pharma companies|Pfizer: Work in a consultancy that works with Pharma companies|Sanofi: Work in a consultancy that works with Pharma companies|Takeda: Work in a consultancy that works with Pharma companies Vaneet Pal Kaur Khurana, Masters in Pharmacy, Merck & Co., Inc.: I work for a consultancy that contracts with Pharma companies|P'fizer: I work for a consultancy that contracts with Pharma companies|Takeda: I work for a consultancy that contracts with Pharma companies|Vasomune Therapeutics: I work for a consultancy that contracts with Pharma companies Prashant Soni, Masters in Pharmacy, BMS: I work in a consultancy that contracts with Pharma companies|Merck: I work in a consultancy that contracts with Pharma companies|Pfizer: I work in a consultancy that contracts with Pharma companies|Sanofi: I work in a consultancy that contracts with Pharma companies|Takeda: I work in a consultancy that contracts with Pharma companies|Vasomune: I work in a consultancy that contracts with Pharma companies Sachin Kumar, TBD, BMS: I work for a company that contracts with Pharma companies|Merck: I work for a company that contracts with Pharma companies|Pfizer: I work for a company that contracts with Pharma companies|Sanofi: I work for a company that contracts with Pharma companies|Takeda: I work for a company that contracts with Pharma companies Emre Yucel, PhD, Merck & Co., Ltd: Stocks/Bonds (Public Company)

